# Inlays, Their Advantages and Limitations

**Published:** 1900-11-15

**Authors:** C. N. Johnson

**Affiliations:** Chicago, Ills.


### INLAYS, THEIR ADVANTAGES AND LIMITATIONS.

BY C. N. JOHNSON, L.D.S., D.D.S., CHICAGO, ILLS.

Read before the National Dental Association, July 10, 1900, and reprinted from the *Dental Cosmos*.

The demand for inlay fillings arises chiefly from two factors which present themselves in the problem of saving the natural teeth when badly affected by caries. The first of these relates to the unsightliness of most metal fillings when exposed to view in the anterior part of the mouth, and the second to the fact that in many instances of extended caries the insertion of foil fillings proves too great a tax on the patient.

The indiscriminate exposure of gold in incisors and cuspids has justly given to American dentistry a certain reputation for vulgar display which can not longer be ignored. If we, as a profession, are to rise to the highest possibilities of our art we must exercise a due regard for the esthetic effects of our work, as well as for its utilitarianism. It has truly been said that the most perfect conception of art is to conceal art, and in no department of human endeavor is this more strikingly true than in the practice of dentistry.

Unfortunately for us and for our patients, we are not yet able with our present facilities to uniformly combine esthetic effects with utility, for, much as the herald of trumpets has sounded forth throughout the land, the dental millennium is not yet upon us.

There are certain cases where, by the judicious use of inlay work, ideal results may be attained, but there are a vast number of others where the conditions are such that inlays can not by any possible means be made artistic and permanent. In alluding to the artistic features of inlay work reference is of course made to porcelain inlays, and it is doubtless true that a properly constructed porcelain inlay is more artistic in appearance than any other kind of filling material. But when this is said, almost all is said that may be in recommendation of this kind of work. The simple fact is that in the very cases where the greatest need is manifest to avoid conspicuous operations porcelain fails in other important requisites to a degree which renders its use almost prohibitory.

For instance, in a long, thin, translucent central or lateral incisor with an approximal cavity which involves the incisal angle we have a type of case which calls not only for harmony of color, but for some material which possesses great strength in a given bulk. It is here, if in any place, that appearance is important, and in no less a degree is it necessary to reproduce the lost angle with a substance which is capable of withstanding attrition and not suffer displacement or fracture. Porcelain, when carried out into thin edges, such as are sometimes called for in these cases, has not the requisite strength or toughness to do extended service in the majority of mouths. It will not do to point to a few isolated instances where porcelain has proved satisfactory in such cases, and argue therefrom that it is always to be depended upon. In some mouths the volume of usage on a given tooth is very small, and any kind of a filling material will do service, but these are the exceptions.

It must be acknowledged that a perfectly condensed gold filling is infinitely tougher and less friable than porcelain, yet even the best inserted gold fillings are sometimes inadequate to the strain. Then the question of anchorage is often an important factor in these fillings. With the incisal angle gone and the necessity existing for its reproduction, a certain area of the inlay is exposed directly to the force of attrition, and the anchorage afforded by the cement can not be considered sufficient to maintain the inlay in place for any extended use. It has been found that in the insertion of gold in these cavities, with all the nicety of adaptation possible, and with the greatest density and strength, failures have too frequently occurred through displacement of the filling from the incisal anchorage, so that observant operators are quite generally resorting to the plan of anchoring these fillings by drilling across the incisal portion of the tooth and making an anchorage at right angles with the approximal cavity. This seems the only certain method of locking these fillings securely in place, and it is a method which can not in ordinary cases be successfully followed with porcelain. Even in those instances where a perfect alignment and perfect form can be given the inlay, the lack of strength at the point where the approximal portion joins the incisal portion is always an element of weakness. The filling material must be especially tough at this point to prevent breakage, and, as has already been intimated, porcelain is too friable to be subjected to this kind of stress.

Another important factor in these cases is the protection of thin enamel. With gold—or better yet, with platinum and gold—a wall of enamel may be adequately protected by building the filling over it to the thickness of one-half a millimeter, while porcelain, to afford any protection whatever, must be laid on in appreciable bulk. At best, porcelain must be considered a poor protection to thin enamel walls, even where it can be successfully built over them.

It will thus be seen that in many of these very conspicuous cases the indications are almost wholly against porcelain inlay work, so far at least as permanence of result is concerned, and in passing it may be well to call attention once more to the possibilities of platinum and gold in this particular class of cases. The shade may be so varied with this material as to take away the extreme yellow luster of gold and produce fillings which are never glaringly conspicuous, and which are immeasurably more reliable and secure than the best possible porcelain inlay. A closer study of this material on the part of the profession will accomplish much in the way of disarming the persistent criticism against display in American dentistry, a fact frequently pointed out by our good friend, Dr. H. J. McKellops.

And yet, as has been stated, there is no metal filling which can quite take the place of porcelain in appearance, and it should be our aim to study the possibilities of porcelain and adapt it to those cases where the indications are favorable to its use.

Such cases are represented by all cavities which are not exposed to attrition, but which are freely exposed to view. Those with a broad opening to the labial—particularly those occurring in the gingival third of the labial surface—may be accounted typical cavities for porcelain inlays. It is here that artistic and serviceable work may be accomplished, provided the operator attains a mastery of shades. This question of shading becomes a matter of very great importance in its relation to artistic results, and this, if there were no other reason, should determine the selection of bodies for such work which fuse at a temperature sufficiently high to assure permanence of color. The bodies which are capable of being fused on gold can not be relied on to permanently maintain their color in the mouth, nor are they so strong in a given bulk as the higher fusing bodies. If porcelain inlays are indicated at all, the very best and strongest and densest porcelain is none too good for the purpose. The present clamor for short-cut methods in this kind of work is calculated to lead to such imperfections as will eventually bring the work into disrepute, and throw doubt on an otherwise legitimate line of practice.

The indications for inlay work, aside from esthetic considerations—viz. the undue tax on the patient in the insertion of large foil fillings—apply particularly to molars and bicuspids. It is here that inlays really may be made to answer the most useful service. In cases where an appreciable portion of the crown is gone inlays of gold may often be inserted to good advantage, and thus save the patient the tedium of a protracted filling operation. These inlays of gold may be used in very many cases where operators have been in the habit of crowning teeth. It is often a nice point to decide where fillings shall stop and crown work begin, but if the operator has a true conception of the possibilities of gold inlays he may frequently save himself any doubt in the matter by employing this ready method of closing the gap between filling and crowning. A tooth with any appreciable portion of the crown missing has in later years quite generally been consigned to excision of the remaining portion for the purpose of inserting an artificial crown, quite irrespective of the condition of the part still standing. This is hardly living up to the highest possibilities of dentistry, because the history of crowns in the years to come will go to prove that the best interests of the patient will be conserved by deferring crown work as long as possible. The serviceability of many a tooth may be prolonged for years by the judicious employment of inlays in those cases where there has been so great a loss of tooth tissue as to make the insertion of a filling injudicious. The tenure of service of the inlay may be logically added to the ultimate life of the tooth, from the fact that when the inlay fails the tooth may then be crowned with as great a promise of future usefulness as if it had been crowned at the outset. In pursuance of our work upon the natural teeth we should have in mind not only the comfort of the patient at the moment, but the greatest ultimate length of service that we can secure for the organs operated on.

One very favorable feature of gold inlay work relates to the adequate protection it affords to weak walls surrounding the cavity. It is in this respect that gold inlays are vastly superior to porcelain inlays. In preparing cavities for gold inlays the frail enamel may be freely ground and extensively beveled with the assurance that the edges of the inlay when built over them will prove an all-sufficient protection against wear or fracture from impact of mastication. In fact, this is one of the cardinal requirements in preparing these cavities, that the margins of the cavity, particularly those in the occlusal region, be very freely beveled so as to admit of a perceptible overlap of the inlay. A degree of bevel may safely be given the enamel for these inlays which, if employed for foil fillings, would prove an element of weakness to the margins.

Another feature connected with these extensive bevels would seem worth mentioning, viz. the fact that the cementing substance used for holding the inlay in place appears to be in a measure protected by the overlying margin of the inlay. If these cases are carefully examined some time after their insertion it will ordinarily be found that the cement is dissolved out for a short distance under the extreme margin of the inlay, but that the dissolving out seems to stop at this point and not penetrate far enough to jeopardize the inlay. This has been noted so frequently that it has proved a matter of considerable encouragement, so far as the permanency of cement is concerned under gold inlays with extensive bevels.

It was formerly the impression of the essayist that the cementing material was invariably an element of weakness in the use of inlays, but a more extended observation would seem to indicate that we have little to fear from this, particularly with these gold inlays and a high-grade quality of cement. It is with inlays, as with every other line of our work, that the real test, after all, is the test of extended practical experience, and no amount of preconceived opinion can have weight against this kind of evidence. So many of these gold inlays have been examined after years of service, nearly all of them presenting this peculiarity, that it would seem impossible to doubt that where due care has been exercised in the adaptation of the inlay and a good grade of cement used little fear need be had as to any serious washing out of the cement.

But as to the relative merits of fillings and inlays in general, it may be stated that in the vast majority of cavities presented for our treatment fillings are infinitely more serviceable than inlays. The requisites for inlay work are in many respects so arbitrary as to prevent the possibility of its being at all universally applicable. A cavity must be cut in such a way that the orifice is more widely extended than the interior, and in many instances this involves the breaking down of strong walls which with ordinary filling operations might be allowed to remain. For instance, in an approximo-occlusal cavity in a bicuspid where the decay has extended well around to the buccal and lingual surface at the gum margin, but where the walls of the cavity are strong and well supported near the occlusal region, it would be a very radical procedure to cut away the buccal and lingual walls parallel with the decay in the gingival region, as would be necessary to properly fit an inlay. It would not only be a tax on the patient, but would materially weaken the tooth. This is only one of the many cases where a perfectly fitted inlay would involve too serious a sacrifice of strong walls, and this may be said to apply as well to incisors as to bicuspids and molars.

There are numerous cavities which may be made to receive a foil filling without any exposure to view, but which if sufficiently opened out to receive an inlay would thereby be rendered conspicuous. In fact, in the majority of cavities which apply to us for treatment, the insertion of an inlay involves the extension of the cavity outlines to a degree unnecessary in an ordinary filling; and while this extension is often a safeguard against a recurrence of decay in bringing the line between inlay and enamel to a point where it is readily kept clean, it sometimes leads to unnecessary exposure of the operation. No method of reproducing lost enamel can ever quite equal in appearance the natural tooth structure, and the less conspicuous our operations are made the nearer we are likely to approach artistic perfection in our work.

One other factor in connection with inlay work relates to the greater expenditure of time necessary where inlays are used than when ordinary fillings are inserted. It is true that the element of time is a minor matter when considering the accomplishment of the highest class of service to our patients, and yet in the daily routine of office practice time becomes important. With a cavity of average size it is conservative to state that a gold filling may be inserted in one-half the time required for an inlay, and the resultant operation will at least prove equally permanent, so that this feature of the case can not well be ignored.

It may therefore be said in conclusion that the legitimate use of inlays is quite narrowly restricted to those exposed surfaces where artistic effects are very important, and to the cases where the tooth tissue is so extensively broken down as to make the insertion of a foil filling too great a tax on the patient. To employ inlays outside of these indications is either to misconstrue their true function, or to let enthusiasm overbalance judgment.

